# Electrokinetic mobility, pH and conductance/conductivity data for aqueous silica and PTFE suspension of controlled composition for selected temperature ranges

**DOI:** 10.1016/j.dib.2019.104354

**Published:** 2019-08-05

**Authors:** Johannes Lützenkirchen, Antun Barišić, Grégory Lefévre, Tajana Begović

**Affiliations:** a Lützenkirchen Institut für Nukleare Entsorgung - INE, Karlsruher Institut für Technologie (KIT), Hermann-von-Helmholtz-Platz 1, 76344, Eggenstein-Leopoldshafen, Germany; b Department of Chemistry, Faculty of Science, University of Zagreb, Horvatovac 102A, HR-10000, Zagreb, Croatia; c PSL Research University, Chimie ParisTech — CNRS, Institut de Recherche de Chimie Paris, 75005, Paris, France

**Keywords:** Surface charge, Inert surface, Silica, Temperature dependence, Point of zero charge

## Abstract

The Data in Brief contains data on the electrokinetic mobility of PTFE and silica particles in aqueous suspensions as a function of pH and temperature. Furthermore, the concomitant conductivities and pH values are reported both for systems in the absence and presence of PTFE particles as a function of temperature and are compatible with the associated research paper “The influence of temperature on the charging of Polytetrafluoroethylene surfaces in electrolyte solutions” (Barisic et al.). The trend of the electrokinetic charging with temperature can be inferred from this for both kinds of particles. The data on the evolution of the pH and the measured conductivities are valuable input for future models that simulate the charge of inert surfaces at variable temperature.

**Table:** Specifications table

Subject area	*Chemistry*
More specific subject area	*Physical and colloid chemistry*
Type of data	*Tables*
How data was acquired	*Laser-Doppler (PALS) device for electrokinetic mobility and conductance (Nanobrook 90 plus PALS, Brookhaven), pH and conductivity electrodes.*
Data format	*Raw data.*
Experimental factors	*Silica and PTFE particles were used as received. Suspensions and solutions were prepared at room temperature with the respective salts and acids. No specific treatment.*
Experimental features	*The Brookhaven PALS device was used to measure the electrokinetic mobility and conductance as a funcition of temperature for a given suspension over a range of temperatures (from room temperature, where the suspension was prepared to lower temperature). The pH and conductivity of suspensions of given salt, acid and particle concentration and solutions containing salt and acid identical to those of the suspensions were measured as a function of temperature starting from low temperature.*
Data source location	*Karlsruhe, Germany*
Data accessibility	*Data is with this article.*
Related research article	*A. Barisic, J. Lützenkirchen, G. Lefevre, T. Preocanin, The influence of temperature on the charging of Polytetrafluoroethylene surfaces in electrolyte solutions, Coll. Surf. A, in press**[1]**.*

**Table:** 

**Value of the data** • The experimental data in combination with each other can be used to infer the variation of the isoelectric point of PTFE particles and silica particles (AEROSIL380) as a function of temperature [1]. • Conductivity data of suspensions and concomitant solutions can be used as quantitative tests of models to describe the charging of PTFE particles as a function of pH and temperature [1]. • The data involve a more comprehensive data set than the currently available one [2] for hydrophobic surfaces. • The data can be used to construct charging models for a range of temperatures and modelers can benefit from the temperature-dependent data. • The data can help to unravel the origin of the charging of inert surfaces in electrolyte solutions, which is currently under debate, and to inspire even more comprehensive experimentation on the effect of temperature.

## 1 Data

The shared data involve the pH, electrophoretic mobility, the conductivity and the conductance of solutions and suspensions (containing particles of interest, i.e. PTFE or silica particles) as a function of temperature. In the experiments, we vary the initial pH of the solution and the salt composition. Table 1, Table 2, Table 3, Table 6, Table 7, Table 11, Table 12 present data on the electrophoretic mobilities and the conductance of the respective suspension for negative, uncharged, and positive surfaces. Table 4, Table 5, Table 8, Table 9, Table 10, Table 13, Table 14 include the corresponding conductivities and pH values. Table 8 a and b show the reproducibility in terms of pH measurements. Table 10 a, b, c, and d show reproducibility in terms of conductivity measurements.

**Table 1:** Electrophoretic mobility (and standard deviation) and conductance of a PTFE suspension in 10 mM NaCl as a function of temperature. At room temperature the pH of the PTFE suspension was 4.

T (K)	μ (μm/s/{V/cm})	σ_μ_ (μm/s/{V/cm})	G (μS)
296.15	−2.31	0.189	2831
294.15	−2.21	0.121	2696
291.15	−2.07	0.083	2519
290.15	−2.02	0.094	2468
287.15	−1.90	0.054	2296
285.15	−1.83	0.079	2181
282.15	−1.67	0.063	2021
279.15	−1.59	0.056	1853
276.15	−1.48	0.072	1694

**Table 2:** Electrophoretic mobility (and standard deviation) and conductance of a PTFE suspension in 10 mM NaCl as a function of temperature. At room temperature the pH of the PTFE suspension was 5.

T (K)	μ (μm/s/{V/cm})	σ_μ_ (μm/s/{V/cm})	G (μS)
293.15	−1.57	0.27	2124
291.15	−1.57	0.25	2061
289.15	−1.48	0.27	1964
287.15	−1.56	0.16	1870
284.15	−1.50	0.14	1730
281.15	−1.31	0.24	1593
278.15	−1.16	0.06	1475
276.15	−1.02	0.13	1387

**Table 3:** Electrophoretic mobility (and standard deviation) and conductance of an Aerosil380 suspension in 10 mM NaCl as a function of temperature. At room temperature the pH of the Aerosil380 suspension was 4.8.

T (K)	μ (μm/s/{V/cm})	σ_μ_ (μm/s/{V/cm})	G (μS)
295.15	−1.02	0.42	3904
293.15	−1.16	0.28	3720
291.15	−1.43	0.23	3550
289.15	−1.64	0.15	3400
286.15	−1.74	0.11	3160
284.15	−1.78	0.08	3015
282.15	−1.82	0.06	2854
280.15	−1.75	0.08	2706
278.15	−1.77	0.06	2569
276.15	−1.72	0.05	2408

**Table 4:** pH of a solution (a) and pH of a PTFE suspension (b) containing 0.1 mM KNO_3_ as a function of temperature. At room temperature the pH of the PTFE suspension was 4.8. The mass concentration in the PTFE suspension was 0.35 g/L.

T (K)	pH
(a)	
282.08	4.655
282.68	4.663
283.11	4.670
283.68	4.677
284.06	4.686
284.58	4.691
285.17	4.700
285.72	4.710
286.24	4.715
286.64	4.724
287.11	4.729
287.64	4.728
288.13	4.737
288.65	4.730
289.25	4.739
289.73	4.753
290.24	4.750
290.74	4.751
291.24	4.751
(b)	
282.11	4.638
282.69	4.643
283.10	4.645
283.63	4.651
284.13	4.660
284.72	4.662
285.17	4.668
285.70	4.669
286.20	4.676
286.67	4.674
287.02	4.666
287.44	4.674
287.92	4.677
288.29	4.676
288.70	4.678
289.03	4.677
289.41	4.682
289.75	4.675
290.08	4.683
290.38	4.688
290.72	4.685
291.01	4.681
291.32	4.689

**Table 5:** Conductivity of a solution (a) and conductivity of a PTFE suspension (b) containing 0.1 mM KNO_3_ as a function of temperature. At room temperature the pH of the PTFE suspension was 4.8. The mass concentration in the PTFE suspension was 0.35 g/L.

T (K)	κ (mS/m)
(a)	
282.08	1.556
282.68	1.574
283.11	1.585
283.68	1.605
284.06	1.642
284.58	1.662
285.17	1.683
285.72	1.698
286.24	1.719
286.64	1.732
287.11	1.755
287.64	1.772
288.13	1.789
288.65	1.806
289.25	1.825
289.73	1.844
290.24	1.861
290.74	1.876
291.24	1.891
(b)	
282.11	1.560
282.69	1.590
283.10	1.613
283.63	1.641
284.13	1.667
284.72	1.696
285.17	1.718
285.70	1.744
286.20	1.769
286.67	1.790
287.02	1.807
287.44	1.826
287.92	1.848
288.29	1.865
288.70	1.884
289.03	1.900
289.41	1.917
289.75	1.933
290.08	1.949
290.38	1.962
290.72	1.978
291.01	1.991
291.32	2.005

**Table 6:** Electrophoretic mobility and conductance of a PTFE suspension in 10 mM NaCl as a function of temperature. At room temperature the pH of the PTFE suspension was 2.5.

T (K)	μ (μm/s/{V/cm})	σ_μ_ (μm/s/{V/cm})	G (μS)
296.15	−0.02	0.09	6009
293.15	−0.01	0.09	5436
289.15	−0.02	0.15	5045
286.15	0.03	0.11	4818
283.15	−0.03	0.14	4614
281.15	0.00	0.09	4512
279.15	−0.03	0.14	4432

**Table 7:** Electrophoretic mobility and conductance of an Aerosil380 suspension in 10 mM NaCl as a function of temperature. At room temperature the pH of the AEROSIL380 suspension was 3.

T (K)	μ (μm/s/{V/cm})	σ_μ_ (μm/s/{V/cm})	G (μS)
297.15	0.06	0.09	3258
294.15	0.04	0.06	2837
291.15	0.03	0.12	2574
289.15	0.03	0.06	2447
286.15	0.04	0.14	2109
283.15	0.03	0.06	1877
281.15	0.02	0.08	1734
279.15	0.04	0.06	1595
276.15	0.05	0.11	1422

**Table 8:** pH of two solutions (a, b) and pH of a PTFE suspension (c) containing 0.1 mM KNO_3_ as a function of temperature. At room temperature the pH of the PTFE suspension was 3.05. The mass concentration in the PTFE suspension was 0.83 g/L.

T (K)	pH
(a)	
278.60	2.964
278.97	2.965
279.68	2.966
280.35	2.970
280.97	2.973
281.55	2.976
282.09	2.978
282.61	2.981
283.35	2.986
283.84	2.989
284.32	2.991
284.76	2.994
285.20	2.996
285.62	2.999
286.20	3.002
286.58	3.004
287.11	3.008
287.61	3.010
288.09	3.012
288.68	3.016
289.23	3.019
289.72	3.022
290.19	3.025
290.73	3.029
291.13	3.033
291.58	3.037
292.00	3.040
292.31	3.043
292.60	3.046
(b)	
278.10	2.957
278.91	2.963
279.69	2.969
280.41	2.974
281.07	2.978
281.69	2.980
282.28	2.985
282.83	2.988
283.35	2.991
283.82	2.995
284.28	2.997
284.91	3.000
285.51	3.002
286.12	3.006
286.72	3.009
287.29	3.013
287.83	3.015
288.34	3.019
288.81	3.022
289.25	3.025
289.81	3.029
290.33	3.032
290.81	3.034
291.25	3.038
291.84	3.042
292.29	3.044
292.70	3.049
(c)	
278.94	3.019
279.31	3.020
279.70	3.024
280.06	3.025
280.77	3.027
281.42	3.030
282.36	3.034
283.19	3.036
283.97	3.040
284.92	3.045
285.76	3.050
286.51	3.054
287.01	3.055
287.46	3.059
288.01	3.061
288.51	3.063
288.96	3.064
289.43	3.066
289.93	3.070
290.43	3.073
291.04	3.077
291.57	3.082
292.06	3.087

**Table 9:** Conductivity of a solution(a) and conductivity of a PTFE suspension (b) containing 0.1 mM KNO_3_ as a function of temperature. At room temperature the pH of the PTFE suspension was 3.05. The mass concentration in the PTFE suspension was 0.83 g/L.

T (K)	κ (mS/m)
(a)	
278.60	36.0
278.97	36.4
279.68	37.0
280.35	37.5
280.97	38.0
281.55	38.5
282.09	38.9
282.61	39.2
283.35	39.9
283.84	40.4
284.32	40.7
284.76	41.1
285.20	41.5
285.62	41.8
286.20	42.3
286.58	42.6
287.11	43.0
287.61	43.4
288.09	43.8
288.68	44.3
289.23	44.8
289.72	45.2
290.19	45.6
290.73	46.0
291.13	46.3
291.58	46.7
292.00	47.0
292.31	47.2
292.60	47.5
(b)	
278.94	34.2
279.31	34.5
279.70	34.8
280.06	35.1
280.77	35.6
281.42	36.1
282.36	36.8
283.19	37.4
283.97	38.0
284.92	38.7
285.76	39.2
286.51	39.9
287.01	40.3
287.46	40.7
288.01	41.1
288.51	41.4
288.96	41.7
289.43	42.1
289.93	42.4
290.43	42.7
291.04	43.2
291.57	43.5
292.06	43.8
292.57	44.2
293.02	44.5

**Table 10:** Conductivity of four solutions (a, b, c, d) and conductivity of a PTFE suspension (e) containing 0.1 mM KNO_3_ as a function of temperature. The room temperature pH of the PTFE suspension was 3.05. The entries in italic in (d) correspond to the suspension, resulting from addition of PTFE particles at about 6 °C PTFE particles (resulting mass concentration 0.83 g/L).

T (K)	κ (mS/m)
(a)	
274.30	32.4
274.57	32.6
274.82	32.8
275.11	33.0
275.39	33.2
275.67	33.5
276.22	33.9
276.72	34.3
277.20	34.7
277.69	35.1
278.14	35.4
278.57	35.8
278.98	36.0
(b)	
280.99	38.0
281.93	38.7
282.74	39.2
283.45	39.9
284.08	40.5
284.64	40.9
285.16	41.4
285.64	41.8
286.09	42.1
286.88	42.8
287.57	43.1
288.19	43.8
288.74	44.1
289.26	44.4
289.93	44.9
290.52	45.4
291.05	45.6
291.64	46.3
292.17	46.8
292.74	47.2
293.23	47.6
(c)	
278.47	35.6
278.88	36.0
279.30	36.3
279.88	36.8
280.43	37.2
280.95	37.6
281.47	38.1
281.97	38.4
282.45	38.8
282.90	39.1
283.49	39.7
283.91	40.0
284.43	40.4
284.93	40.8
285.41	41.2
285.85	41.5
286.38	41.9
286.88	42.3
287.43	42.7
287.88	43.1
288.39	43.5
288.87	43.9
289.31	44.2
289.80	44.6
290.19	44.9
290.61	45.2
291.00	45.5
291.31	45.7
291.64	45.9
291.99	46.2
292.35	46.4
292.64	46.6
292.90	46.8
293.15	46.9
(d)	
275.49	33.1
276.18	33.8
276.83	34.3
277.45	34.8
278.03	35.3
278.58	35.7
279.11	36.1
279.60	36.5
280.08	36.9
280.55	37.3
281.14	37.8
281.57	38.1
282.12	38.5
282.64	38.9
283.14	39.2
283.73	39.8
284.29	40.3
284.93	40.8
285.43	41.2
285.91	41.5
286.44	42.0
287.03	42.4
287.50	42.8
287.93	43.1
288.35	43.4
288.82	43.7
289.30	44.0
289.75	44.3
290.16	44.6
290.59	44.9
290.95	45.1
291.32	45.4
291.66	45.6
292.00	45.8
292.42	46.1
292.71	46.3
(e)	
275.94	33.7
276.47	34.1
276.98	34.5
277.45	34.9
277.91	35.3
278.37	35.6
278.77	35.9
279.42	33.1
280.13	33.7
280.81	34.2
281.48	34.7
282.12	35.2
282.73	35.6
283.30	36.1
283.84	36.5
284.35	36.9
284.85	37.2
285.32	37.6
285.76	37.9
286.19	38.2
286.59	38.5
286.98	38.8
287.34	39.0
288.19	39.7
288.50	39.9
289.02	40.3
289.50	40.7
289.87	41.0
290.40	41.3
290.87	41.7
291.30	42.0
291.69	42.3
292.04	42.6
292.35	42.8
292.73	43.1
293.00	43.3
293.21	43.5

**Table 11:** Electrophoretic mobility (and standard deviation) and conductance of a PTFE suspension in 10 mM NaCl as a function of temperature. At room temperature the pH of the PTFE suspension was 1.8.

T (K)	μ (μm/s/{V/cm})	σ_μ_ (μm/s/{V/cm})	G (μS)
296.15	0.21	0.10	19172
294.15	0.16	0.06	18324
291.15	0.14	0.24	17389
287.15	0.07	0.09	16115
285.15	0.03	0.05	15251
283.15	0.03	0.09	14674
280.15	0.06	0.12	13592
278.15	0.04	0.09	12860
276.15	0.02	0.07	12123

**Table 12:** Electrophoretic mobility (and standard deviation) and conductance of an Aerosil380 suspension in 10 mM NaCl as a function of temperature. At room temperature the pH of the Aerosil380 suspension was 2.7.

T (K)	μ (μm/s/{V/cm})	σ_μ_ (μm/s/{V/cm})	G (μS)
297.15	0.38	0.08	3683
294.15	0.35	0.06	3387
292.15	0.32	0.07	3210
289.15	0.31	0.04	2939
287.15	0.29	0.04	2799
285.15	0.28	0.07	2653
283.15	0.27	0.06	2530
279.15	0.27	0.06	2270
276.15	0.25	0.04	2084

**Table 13:** pH and conductivity of a solution (a) and conductivity of a PTFE suspension (b) containing 0.1 mM KNO_3_ as a function of temperature in lower temperature ranges. The experiment was started at 8 °C and measurements were stopped at about 15 °C. At room temperature the pH of the PTFE suspension was 1.65. The mass concentration in the PTFE suspension was 0.5 g/L.

T (K)	pH	κ (mS/m)
(a)		
281.36	1.490	677.9
282.88	1.538	695.3
284.29	1.566	713.5
285.26	1.582	726.5
286.07	1.592	737.0
286.80	1.599	745.9
287.48	1.605	753.9
(b)		
281.04	1.543	767.1
282.26	1.566	787.2
283.28	1.580	803.6
284.15	1.590	817.7
284.97	1.598	830.5
285.73	1.605	842.2
286.44	1.617	852.7

**Table 14:** pH and conductivity of a solution (a) and pH and conductivity of a PTFE suspension (b) containing 0.1 mM KNO_3_ as a function of temperature in lower temperature ranges. The experiment was started at 17 °C and measurements were stopped at room temperature. At room temperature the pH of the PTFE suspension was 1.65. The mass concentration in the PTFE suspension was 0.5 g/L.

T (K)	pH	κ (mS/m)
(a)		
290.26	1.599	785.5
291.12	1.608	794.9
291.88	1.614	803.0
292.53	1.619	810.2
293.11	1.623	816.5
293.62	1.627	822.1
294.24	1.632	829.1
(b)		
290.34	1.619	907.9
291.15	1.624	919.2
291.86	1.628	928.4
292.48	1.632	936.8
293.04	1.634	944.7
293.52	1.638	951.4
294.17	1.642	961.8

**Fig. 1:**
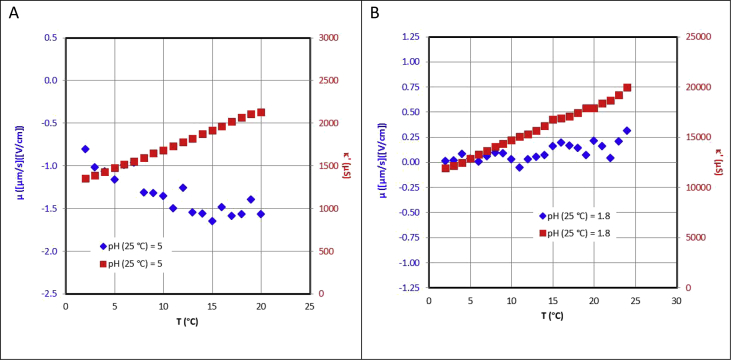
a) Electrophoretic mobility and conductance of a PTFE suspension in 10 mM NaCl as a function of temperature. At room temperature the pH of the PTFE suspension was 5. b) Corresponding plot for a net positive surface, where the pH of the suspension at room temperature was 1.8.

Fig. 1a shows an example pertaining to Table 2 for a negatively charged surface, while Fig. 1b shows the same kind of data for the positively charged surface (Table 11). The tables include the relevant experimental error estimates.

The trends in electrophoretic mobility are inversed based on the average values.

## 2 Experimental design, materials, and methods

The electrokinetic mobilities were measured using the Brookhaven Zeta-PALS equipment. This set-up also measures conductance. The settings in most cases were such that the temperature was varied from room temperature down to low temperatures. In some cases, it was also chosen to start an experiment directly at a lower temperature to observe whether the starting point would affect the results. These experiments were done both with PTFE particles and AEROSIL380 particles.

Separate pH and conductivity measurements were obtained using the SurPass equipment. The pH and conductivity were measured starting at low temperature and letting the temperature slowly increase to room temperature. This was done for defined solutions in terms of salt and acid composition both with and without PTFE particles of known mass. In some experiments, a known mass PTFE particles were added to a solution with known volume (at room temperature) that had reached a given temperature.

PTFE particles were Microdispers-200 obtained from Polysciences Europe (Hirschberg, Germany). AEROSIL380 particles were obtained from EVONIK (Germany). All chemicals were reagent grade. Solutions were made using MilliQ water.

The pH and conductivity electrode of the SurPass equipment were calibrated as described in the manual of the set-up. The data were collected in the premeasurement option “record”.

The data are all presented as the raw data in terms of tables.
